# Proteomics Reveals Novel *Drosophila* Seminal Fluid Proteins Transferred at Mating

**DOI:** 10.1371/journal.pbio.0060178

**Published:** 2008-07-29

**Authors:** Geoffrey D Findlay, Xianhua Yi, Michael J MacCoss, Willie J Swanson

**Affiliations:** Department of Genome Sciences, University of Washington, Seattle, Washington, United States of America; Duke University, United States of America

## Abstract

Across diverse taxa, seminal fluid proteins (Sfps) transferred at mating affect the reproductive success of both sexes. Such reproductive proteins often evolve under positive selection between species; because of this rapid divergence, Sfps are hypothesized to play a role in speciation by contributing to reproductive isolation between populations. In *Drosophila*, individual Sfps have been characterized and are known to alter male sperm competitive ability and female post-mating behavior, but a proteomic-scale view of the transferred Sfps has been missing. Here we describe a novel proteomic method that uses whole-organism isotopic labeling to detect transferred Sfps in mated female D. melanogaster. We identified 63 proteins, which were previously unknown to function in reproduction, and confirmed the transfer of dozens of predicted Sfps. Relative quantification of protein abundance revealed that several of these novel Sfps are abundant in seminal fluid. Positive selection and tandem gene duplication are the prevailing forces of Sfp evolution, and comparative proteomics with additional species revealed lineage-specific changes in seminal fluid content. We also report a proteomic-based gene discovery method that uncovered 19 previously unannotated genes in D. melanogaster. Our results demonstrate an experimental method to identify transferred proteins in any system that is amenable to isotopic labeling, and they underscore the power of combining proteomic and evolutionary analyses to shed light on the complex process of *Drosophila* reproduction.

## Author Summary

Across many species, males transfer both sperm and seminal proteins to their mates. These proteins increase male reproductive success by improving sperm competitive ability and modifying female behavior. In *Drosophila*, seminal proteins increase female rates of egg-laying and sperm storage and reduce a female's willingness to mate with subsequent suitors. Several male seminal proteins have been extensively characterized, and others have been predicted based on gene expression patterns, yet the full set of proteins that is transferred to females has not been defined. Here we introduce a new proteomic method that identifies transferred seminal proteins in recently mated females and quantifies their relative abundance. We confirm many of the predicted seminal proteins and discover a number of novel seminal fluid components. Some of these proteins show elevated rates of evolution, consistent with their involvement in sexual selection or sexual conflict, and many have arisen by tandem gene duplication. By using this method in three species of *Drosophila*, we identified lineage-specific components of seminal fluid. Additionally, we developed and validated a method to identify completely new genes in the D. melanogaster genome. These transferred proteins are now targets for follow-up genetic, biochemical, and evolutionary analysis.

## Abstract 3

Seminal fluid proteins are critical for male reproductive success. This paper describes a new method to identify these proteins in recently mated females and details their dynamic evolutionary histories.

## Introduction

In addition to sperm, males of many internally fertilizing species transfer seminal fluid proteins (Sfps) to their mates during copulation. These proteins function in a variety of reproductive processes, including sperm capacitation, sperm storage and competition, and fertilization, and in some cases they affect female behavior and physiology [1]. Like other reproductive proteins, Sfps often evolve rapidly between species, underscoring their relevance to reproductive success [2]. Sfps are thought to interact with several classes of molecules, including other Sfps (which may originate from seminal fluid of the same male or from a competitor), proteins native to the female reproductive tract, and pathogens that may be introduced during the course of mating. These interactions create opportunities for coevolution, leading to speculation that sperm competition, sexual conflict, sexual selection, and/or host–pathogen interactions could drive the rapid, adaptive evolution of many Sfps [3]. Because of their rapid evolution and their critical importance to reproductive fitness, Sfps may also be involved in the formation of new species [3–5]. As such, researchers have sought to identify and characterize Sfps in such diverse taxa as mosquitoes, crickets, honeybees, rodents, and primates [6–10].

Although Sfps are being studied in many species, they are best characterized in Drosophila melanogaster. Because the *Drosophila* mating system features multiple matings by females and sperm competition between males [11–14], Sfps are thought to be especially important for reproductive success and for mediating conflict and competition. Previous studies have focused on three areas: (a) the effects of the full set of Sfps (and especially of accessory gland proteins, or Acps) on male and female fitness; (b) identification of putative Acps by expressed sequence tag (EST) sequencing, comparative genomics, and proteomics; and (c) functional analysis of specific Acps. Whole-organism work revealed that Acps mediate a “cost of mating” to females. The repeated receipt of Acps through multiple matings lowers female reproductive output by reducing female life span without a corresponding increase in egg production [15–17]. Furthermore, when a population of males harboring natural genetic variation was allowed to adapt to a static female genotype, male sperm competitive ability, mating success, and harm caused to females increased in 30–40 generations [18]. These dramatic evolutionary outcomes sparked much interest in identifying the specific proteins of seminal fluid. Screens for genes expressed specifically in the male accessory glands (and encoding proteins predicted to be secreted) identified ∼70 putative Acps [19,20], several of which have been genetically or biochemically characterized (reviewed in [21]). Work in related *Drosophila* species has revealed that many predicted Acps are subject to lineage-specific gene gain, gene loss, and/or copy number variation [22–24]. Additionally, several proteomic studies have examined both Acps and sperm proteins found in males [25,26], and whole-genome, tissue-specific microarray analysis has increased the number of predicted Acps to 112 [21,27].

In spite of this considerable progress, less than one-third of the predicted D. melanogaster Sfps have been detected in mated females [21,28]. Furthermore, prior work to predict Sfps has often required that candidate genes show tissue-specific expression in the male reproductive tract. Identifying the set of transferred Sfps in an unbiased fashion is of critical importance, since it is these proteins that are the most likely to influence post-mating processes like sperm competition and sexual conflict. We have developed a mass spectrometry (MS) method that specifically detects male Sfps in mated female D. melanogaster. In addition to confirming the transfer of many predicted Sfps, we identified dozens of new seminal fluid components, including completely new classes of proteins. Evolutionary analyses show that positive selection and tandem gene duplication drive the evolution of seminal fluid between species, and comparative proteomics with additional species identified lineage-specific Sfps. We also used our MS data to estimate the relative abundance of each Sfp in seminal fluid and to discover previously unannotated genes encoding additional Sfps. Taken together, our experiments illustrate the power of combining proteomics with evolutionary biology to address fundamental questions about reproduction.

## Results

### Isotopic Labeling of Female Flies Allows Transferred Sfps to Be Identified

To distinguish between transferred Sfps and proteins native to the female reproductive tract, we metabolically labeled female flies using a diet enriched in ^15^N isotopes to create an isotopically “heavy” form of the female proteins [29]. Females were reared on yeast that was grown in media enriched in ^15^N. After one full generation of labeling, the ^15^N enrichment in detected fly peptides was ∼98 atom percent excess, and no peptides from whole female flies were identified with natural abundance nitrogen isotopes. These data confirm that isotopic labeling can be readily achieved in D. melanogaster and other drosophilids (see below). Therefore, we reasoned that by mating unlabeled males to labeled females and then analyzing proteins found in the female reproductive tract by MS, transferred male Sfps could be identified by those peptides that showed natural abundance isotope distributions. We chose to label females instead of males, because MS resolution is best for unlabeled peptides, and we were interested in identifying male Sfps.

We performed multiple biological replicates of mating experiments with different strains of males: Canton S (a standard lab strain), sons of *tudor* females (spermless males) [30], and, as a negative control, DTA-E males (which are spermless and do not produce main cell accessory gland proteins) [31]. In two DTA-E experiments, we detected 11 transferred proteins, including several known to be produced outside of the accessory glands (Table S1). Six total experiments with Canton S and *tudor* males identified a set of 138 high-confidence Sfps (Table 1 and Table S2), using a peptide-level *q*-value ≤ 0.01 within each experiment [32]. Just over half (75/138) of the transferred Sfps were previously predicted through tissue-specific expression profiling or other experimental or comparative genomic methods [21], but only 19 were confirmed previously to be transferred at mating. Notably, we found only five previously documented sperm proteins [25], confirming that our protein preparation protocol effectively selected for soluble, extracellular proteins. We did not detect 49 predicted Sfps [21]. These proteins may be transferred at low levels, immediately cleaved or degraded in the female, or have certain peptide sequences or post-translational modifications that complicate detection by shotgun proteomics. Alternatively, some may not be transferred at mating.

**Table 1:**
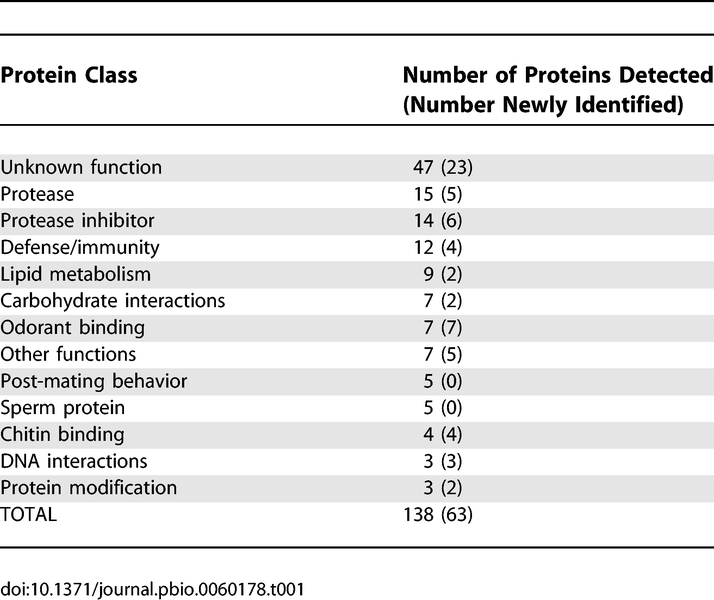
Classes of Seminal Fluid Proteins Detected by MS in Recently Mated D. melanogaster Females

We identified 63 novel Sfps, 45 of which were found in at least two biological replicates. Many of these proteins fell into the same functional categories as the previously predicted set, including proteases, protease inhibitors, mediators of an immune response, and proteins involved in lipid metabolism (Table 1). We discovered several new classes of proteins among the transferred Sfps. Most intriguing were six members of the odorant binding protein (Obp) family [33]. Obps are thought to shuttle small molecules through aqueous solutions by binding them in a small, hydrophobic pocket; they are traditionally associated with the olfactory nervous system [34]. We confirmed that these Obps are transferred in seminal fluid by performing MS on protein digests from dissected accessory glands and by confirming each gene's expression in the male reproductive tract with FlyAtlas [27].

### Positive Selection and Tandem Gene Duplication Drive the Evolution of Sfps

**Figure 1:**
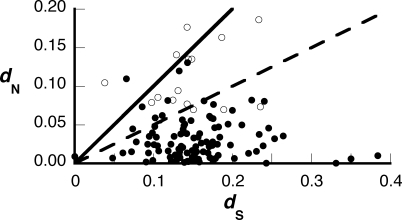
Whole-Gene, Pairwise Estimates of *d*_N_ and *d*_S_ for Transferred Sfps of D. melanogaster Each D. melanogaster Sfp was aligned to its ortholog in either D. simulans or D. sechellia, and a pairwise, whole-gene estimate of each rate was made. Those genes with ω > 0.5 (dashed line) and/or *d*_N_ > 0.05 were analyzed for sites under selection using multiple species alignments from the genome sequencing project. Open dots represent genes with significant evidence for positive selection acting on a subset of sites in the M8 versus M8a model comparison in *codeml* (see Table S3). The solid line represents ω = 1.

Reproductive proteins of diverse species often evolve under positive Darwinian selection, which may indicate involvement in a coevolutionary process such as sexual selection, sexual conflict, or host–pathogen recognition [2]. We used coding sequence alignments from the 12 *Drosophila* genomes project [35,36] to calculate the rates of nonsynonymous substitution (*d*_N_) and synonymous substitution (*d*_S_) for all Sfps for which an ortholog was identified (116 of the total 138). For each Sfp, we determined the whole-gene, pairwise *d*_N_/*d*_S_ (ω) ratio between the D. melanogaster gene and an ortholog from a closely related species (Figure 1). By this conservative test, five Sfps showed evidence of adaptive evolution (ω > 1). However, prior studies have shown that when the whole-gene pairwise ω ratio exceeds 0.5, or when the nonsynonymous substitution rate (*d*_N_) is elevated, there are often specific sites within the protein for which adaptive evolution can be detected with more sensitive methods [7,37]. Therefore, we used multiple species alignments to search for specific residues under selection for all genes with pairwise ω > 0.5 and/or pairwise *d*_N_ > 0.05. (We did not test all Sfps, in order to minimize the number of statistical tests.) We found evidence for adaptive evolution at specific sites for 16 of 36 proteins (Figure 1 and Table S3), including four proteins that were unidentified previously as Sfps. Nine of these tests for selection remained significant after applying a strict Bonferroni correction for multiple tests. These rapidly evolving proteins are attractive targets for future study.

**Figure 2:**
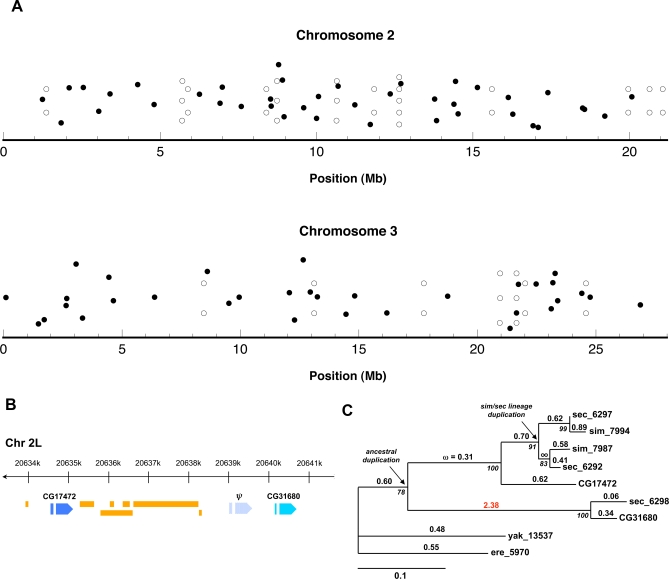
Transferred Sfps Cluster in Small Groups throughout the D. melanogaster Autosomes (A) Euchromatic genomic locations of the autosomal Sfps are shown, based on the version 5.2 genome assembly. Open points indicate Sfps that occur in a cluster, filled points indicate those that do not. We observed significantly more clusters of genes than were found when randomly selecting 138 genes with the observed chromosomal distribution (1,000 simulations; median number of clusters: 3; range: 0–9). (B) Genomic map of Chromosome 2L containing the tandem duplicates *CG17472* and *CG31680*. *CG17472*, which is present in all five species of the *melanogaster* subgroup, contains several residues under positive selection. Orange bars indicate naturally occurring transposon remnants. A pseudogenized copy of the locus is also indicated (ψ). (C) Phylogenetic analysis of the tandem gene duplication. Numbers above branches are branch estimates of ω in the free-ratio model. Italicized numbers under nodes indicate percent bootstrap support based on 1,000 replicates. Numbers at the tips of the non-*melanogaster* branches of the tree are the GLEANR-predicted gene numbers for the indicated species.

Previous studies found that some predicted Sfps are clustered throughout the genome [21,24]. We examined the chromosomal locations of the transferred Sfps and found similar patterns. We defined a cluster as genes with start codons located within 10 kb of each other. We identified 19 clusters of 2–5 transferred Sfps, which contain one-third (46/138) of the detected Sfps (Figure 2A). For 17 clusters, all member genes are transcribed in the same direction, and 15 clusters contain genes that encode proteins with full-length homology to one another. Thus, most of the observed clustering can be attributed to tandem gene duplication. Four paralogous clusters contain at least one gene that was under selection in the sites analysis above. Previous work also found a dearth of Acps on the X chromosome. Consistent with this finding, the 13 transferred Sfps on the X chromosome were significantly fewer than would be expected by chance (χ^2^ = 4.68, 1 degree of freedom [df], *p* = 0.03), given the proportion of annotated genes on the X.

One example of rapidly evolving tandem duplicates is the gene pair *CG17472* and *CG31680*. Across five species, *CG17472* has evolved adaptively, with 21.3% of sites predicted to be under positive selection (estimated ω = 3.36, PAML M8 versus M8a comparison: χ^2^ = 15.38, 1 df, *p* < 0.0001). These duplicates flank a transposition hot spot and a third, pseudogenized copy of the locus (Figure 2B). Additionally, *CG17472* has duplicated along the lineage leading to D. simulans and D. sechellia (Figure 2C). Examining the ω ratio on each branch of the phylogeny reveals a burst of positive selection on the *CG31680* lineage immediately after duplication. Indeed, a branch model allowing for variable selective pressures along each branch (shown in Figure 2C) fit the data significantly better than a model with a uniform ω for all branches (χ^2^ = 29.04, 14 df, *p* = 0.01).

### Relative Quantification Confirms the Importance of the Newly Identified Sfps

**Figure 3:**
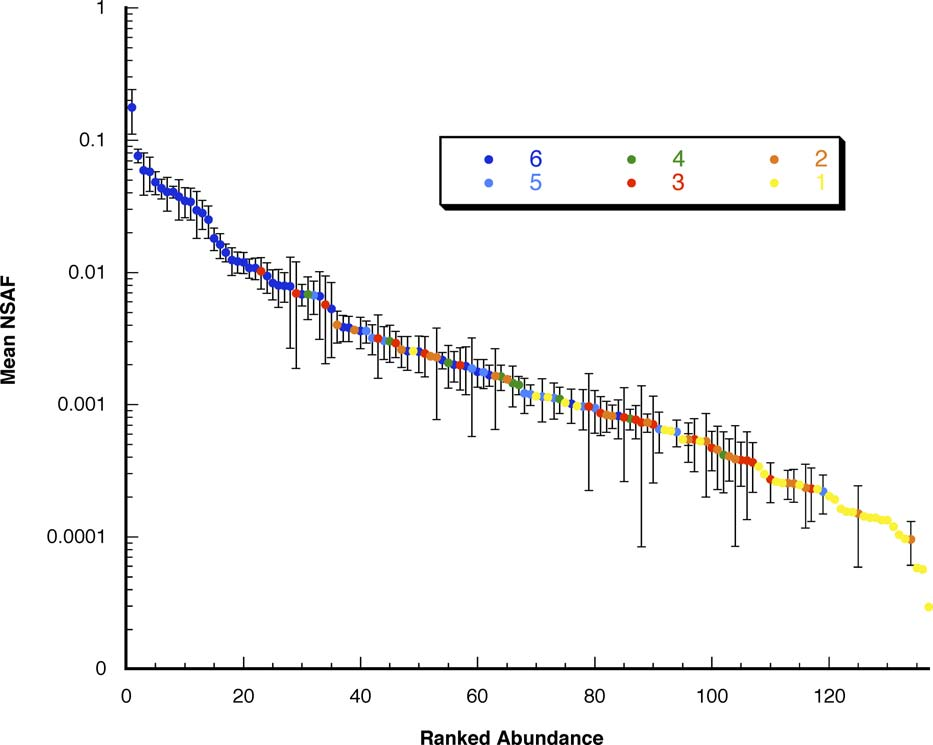
Relative Quantification of Transferred Sfps Found in Mated Female D. melanogaster in a Total of Six Canton S and *tudor* Experiments The NSAF was used to estimate the relative abundance for each identified Sfp within each mating experiments, and values for each protein were averaged across all experiments in which the protein was found. Proteins were sorted by abundance and plotted to show the dynamic range of proteins detected (about 10^3^). The color of each point indicates the number of experiments in which the protein was identified; note that sperm-specific proteins could only be found in a maximum of three experiments (Canton S). Error bars represent one standard error of the mean NSAF.

We used our MS data to estimate the relative abundance of each Sfp in seminal fluid. By counting the number of spectra associated with each Sfp in a given experiment and standardizing by the length of the protein and the total number of Sfp spectra detected in the experiment, we calculated a normalized spectral abundance factor (NSAF) [38,39] for each protein, which could then be averaged across all experiments (Figure 3 and Table S2). Notably, NSAF values were positively associated with the number of biological replicates in which a protein was found (Figure 3). Several of the most abundant proteins were previously characterized Sfps, such as Acp62F (a protease inhibitor) and Acp70A (the sex peptide). However, several novel proteins were also in the top quartile for abundance, including Obp56f, Obp56g, and the tandem duplicate CG17472. Although these NSAF measurements are only approximate, these data provide the first proteomic-scale view of the relative amount of each transferred Sfp, which may be useful for selecting candidates for further investigation.

### Comparative Proteomics Reveals Lineage-Specific Changes in Seminal Fluid Content

To examine the cross-species evolution of seminal fluid content, we used the predicted protein annotations of D. simulans and D. yakuba [35,36] to repeat our mating experiments with a wild-type strain of each species (Figure S1). Of the 63 Sfps detected in all three species, 19 were not reported previously as seminal fluid components. For Sfps that were detected in only one or two species, we investigated whether these proteins could be called as either lineage-specific gene gain or loss events. Most of the proteins had identifiable orthologs in the other species; our failure to detect these proteins may be due to changes in expression patterns, sequence substitutions that render MS identification more difficult, changes in the amounts of proteins transferred at mating, or the lower number of replicates (two per species) performed for D. simulans and D. yakuba. However, our data identify 13 lineage-specific Sfps across the three species (Table S5). For example, in D. melanogaster, CG6289 (a predicted serine protease inhibitor) has duplicated to form the lineage-specific gene CG6663. Also, in *D. yakuba,* Acp76A (another serine protease inhibitor) has duplicated, and several other proteins appear to be either lineage-specific to D. yakuba or rendered nonfunctional in other species (Table S5).

Some proteins detected for D. simulans and D. yakuba lacked annotated orthologs in D. melanogaster. For seven such proteins, we identified the syntenic region in D. melanogaster and performed reverse-transcriptase PCR (RT-PCR) to determine whether transcripts of the region were made. In five cases, we detected a transcript in D. melanogaster (see Table S5), and three of these putative loci were detected as proteins in D. melanogaster when searching for unannotated proteins in the D. melanogaster genome (see below). Curiously, one of these genes, which we have annotated as *Sfp53D*, showed male-specific expression in D. yakuba and male-biased expression in D. simulans, but no sex expression bias in D. melanogaster (data not shown). Sfp53D is therefore an example of the type of protein that would have been omitted from previous sets of predicted Sfps due to its lack of sex-specific expression.

### A Novel Proteomic Method Identifies Unannotated Reproductive Genes in D. melanogaster

Based on these results, we reasoned that other Sfps may not be annotated as genes in *D. melanogaster*, which would make them impossible to detect by searching mass spectra against the annotated proteome. To detect additional unannotated Sfps, we first constructed a six–reading frame translation of the D. melanogaster euchromatic genome, which produced >5.8 million potential open reading frames (ORFs). Then, to reduce computational search time, we applied the Hardklör algorithm [40] to predict which MS2 spectra from a *tudor* experiment came from male peptides containing only natural abundance isotopes. These spectra were searched against the six-frame database, and those that matched an ORF corresponding to an annotated protein were discarded. This procedure left 23 novel, putative ORFs that did not match any D. melanogaster gene annotation in FlyBase. For each putative ORF, we used rapid amplification of cDNA ends (RACE) and RT-PCR to confirm transcription of the region encoding the peptide and to define the full-length transcript. Through this method, we discovered 19 unannotated genes (Table S6; GenBank (http://www.ncbi.nlm.nih.gov/Genbank/) accession numbers EU755332–EU755350), most of which showed no significant identity to the predicted proteins of the other sequenced *Drosophila* species.

**Figure 4:**
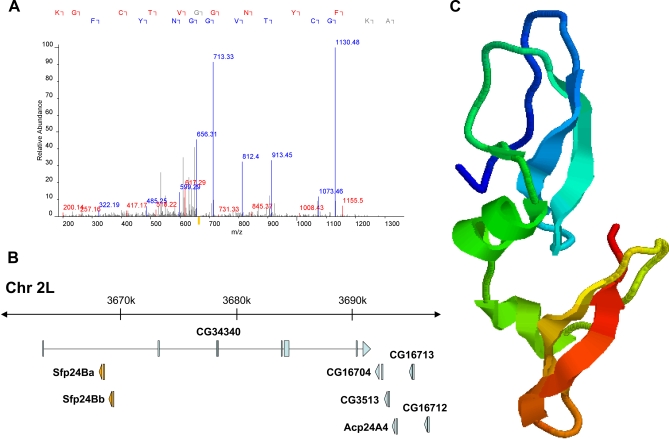
Discovery of Unannotated Genes in D. melanogaster Using a Six–Reading Frame Database Search and RACE (A) Mass spectrum identifying one of three peptides (AKGCTVGGNYFR) detected for Sfp24Ba. Numbers above the peaks indicate mass/charge (*m/z*) ratios used to identify individual residues. Red color indicates *b* ions, blue color indicates *y* ions. (B) Genomic map of Chromosome 2L showing a cluster of two newly identified Sfps (orange) and other annotated genes in the region (blue), including Acp24A4, a transferred Sfp. (C) Predicted three-dimensional structure of Sfp24Ba (with the SignalP-predicted signal sequence removed), which shows significant homology to bikunin and Kunitz-type protease inhibitors (PHYRE *e*-value = 3.1 × 10^−14^). The N and C termini are indicated by blue and red shading, respectively.

All 19 proteins have predicted signal sequences for secretion; many are encoded by only one or two exons, and all produce short polypeptides (median length: 93 residues). Consistent with our clustering analysis, half of the genes were found in regions of the genome containing other annotated Sfps. Most of the novel proteins had no recognizable domains based on BLAST and structural homology searches, but we identified one C-type lectin and three enzyme inhibitors, including a putative protease inhibitor, Sfp24Ba. This protein was identified by three peptides, one of which is indicated in Figure 4A. *Sfp24Ba* is adjacent to another previously unannotated gene, *Sfp24Bb* (an apparent tandem duplicate), and lies 25 kb upstream of the gene that encodes a transferred protease inhibitor, *Acp24A4* (Figure 4B). Comparative structural modeling (Figure 4C) suggests that this protein is a Kunitz-type protease inhibitor. The discovery of these 19 new Sfp genes in a model system that has been studied for over a century and for which comparative genomic analysis is now straightforward underscores the limitations of both computational gene prediction programs and the “whole-proteome” databases that are routinely used during shotgun MS analyses.

## Discussion

Our study provides a proteomic-scale view of the transferred Sfps in D. melanogaster. While we confirmed that 75 predicted Sfps are truly transferred at mating, we also identified a total of 82 genes (63 already annotated, 19 newly discovered) previously unknown to encode seminal fluid products. By using data from the genome sequencing projects and by performing comparative experiments in D. simulans and D. yakuba, we identified many instances of positive selection, tandem gene duplication, and lineage-specific changes in seminal fluid content between species. Taken together, our experiments demonstrate how new proteomic methods can be combined with the vast amounts of genomic sequence data that are now available to gain considerable insight into the molecular players of a specific biological process.

The two methodological advances presented here—the use of isotopic labeling to distinguish between the sexes, and searching MS data against an entire translated genome—should be applicable to many taxa. For example, worms, plants, rodents, and microorganisms are all amenable to isotopic labeling [29,41,42]. In any of these systems, differential labeling should readily allow the detection of proteins transferred from one organism (during mating or another behavior, e.g., courtship). Thus, our approach allows transferred proteins in a pre-specified biological process to be identified. Furthermore, our MS- and RACE-based method to identifying novel genes should be applicable to other organisms with sequenced genomes, particularly if their genome sizes are no more than 1–2 orders of magnitude greater than the D. melanogaster genome. Rodents, *Arabidopsis*, and humans all fall within this range; indeed, recent work in A. thaliana has found new genes using a similar approach [43]. Our results confirm that searching MS data against an entire translated genome, rather than only an annotated or predicted proteome, can identify a considerable number of new genes. Admittedly, this process might have been particularly useful for identifying *Drosophila* Sfps. As shown here and in previous work, these proteins are short, rapidly evolving, and relatively free of codon bias [19,22], three features making them less likely to be detected by computational gene prediction programs. Nonetheless, our gene identification method was straightforward to perform, and because it was experimentally based, it offered automatic verification for the new genes and allowed us to immediately assign them to a specific biological process: male reproduction.

One striking result from these experiments is that seminal fluid content in D. melanogaster appears to be considerably more complex than was previously predicted. The Obp genes identified reproducibly and at high abundance by MS are particularly attractive targets for further characterization. One hypothesis for the origin of these reproductive Obps is based on the fact that Obps are a large, 51-member family in D. melanogaster [33]. If some members of this family were functionally redundant, selection on the regulatory and coding sequences of some Obps might have been relaxed, allowing them to be co-opted from an olfactory function into a male reproductive function. Indeed, several of the identified Obps show accessory gland–specific patterns of expression, while others are expressed in both the accessory glands and the head [27]. The function of these reproductive Obps remains to be determined; they may present odorants or pheromones to odorant receptors in the female reproductive tract or play some other role, such as transferring small molecules to the female to elicit a behavioral response. If some of the Obps interact with a receptor in the female tract, the Or10a odorant receptor is one possible target, since its expression is up-regulated in female reproductive tracts in response to the receipt of Sfps [44].

While the selective pressures driving the evolution of Sfps (and of reproductive proteins in general) remain unclear, the important roles of tandem gene duplication and positive selection in the evolution of Sfps are consistent with the predictions made by models of sexual selection/conflict [45]. If males are engaged in a coevolutionary chase with females, driven by sexual selection or sexual conflict, duplication of an Sfp locus could allow males to better adapt to a particular allele or paralog of a female receptor [45]. Indeed, gene duplication followed by positive selection has been observed previously in a well-characterized reproductive protein, lysin, which allows abalone sperm to penetrate the egg vitelline envelope [46]. If *Drosophila* Sfps are coevolving with receptors in the female reproductive tract—or with other Sfps with which they interact—then gene duplication may be an important evolutionary strategy for males to increase their reproductive success. Tests of this hypothesis will require both functional data on the newly identified male proteins and the identification of their interacting female and/or male partners [47–49].

The rapid divergence characteristic of many Sfps has generated considerable interest in their potential role in speciation [3,4]. If proteins mediating processes such as sperm storage, fertilization, or post-mating behavior diverged quickly between allopatric populations, driven continually by coevolutionary forces such as sexual selection or sexual conflict, between-population matings may become less productive than within-population matings. Such a difference could exert pressure to further differentiate the mating systems or mating behaviors of each group, which could eventually lead to the formation of distinct species. Determining the transferred Sfps, and subsequently identifying their functions and evolutionary patterns, could therefore be important steps in identifying potential “speciation genes.”

In conclusion, this set of transferred proteins provides a rich resource for investigating long-standing evolutionary questions and for identifying the specific molecules and functional allelic variants that affect both sperm competition and male-female coevolution and conflict. The challenge ahead will be to apply the combination of genetic, biochemical, and evolutionary methods that have already yielded many insights into *Drosophila* reproduction to this novel collection of transferred proteins. Functional tests of individual Sfps are essential for understanding the causes of the dramatic post-mating changes in female behaviors. For example, several studies have used gene knockouts or RNA interference to identify the post-mating effects of specific Sfps [50–55]. Other experiments have associated naturally occurring variants in several Sfps with different measures of sperm competition [12,56]. We expect that both of these approaches will become more effective in the future, since they can now be targeted to those transferred Sfps identified here.

## Materials and Methods

### Flies.

Fly stocks were maintained on standard media at 25 °C, except during isotopic labeling (see below). D. melanogaster stocks included a wild-type lab strain, Canton S, and the strain used for genome sequencing, *y*; *cn bw sp*. To produce spermless males, homozygous *tud* ^1^ *bw sp* females [30] were mated to either Canton S or *y*; *cn bw sp* males, and male progeny were retained for use in mating experiments. The DTA-E stock was used to produce males lacking both sperm and main-cell accessory gland proteins [31]. D. simulans strain W89 and D. yakuba strain Tai6 were used in additional mating experiments.

### Isotopic labeling.

The isotopic labeling procedure followed a previously described method [29], with some modifications. Wild-type Saccarhomyces cerevisiae was grown to saturation in minimal media containing 2% glucose, yeast nitrogen base without amino acids and ammonium sulfate (Difco), and ^15^N-labeled ammonium sulfate (≥ 99% ^15^N-enrichment; Spectra Stable Isotopes). Yeast cells were pelletted, resuspended in a small volume of sterile water, and lyophilized. This dried yeast was then mixed with water to form a “heavy” (^15^N) yeast paste. Flies were isotopically labeled by allowing unlabeled females to lay eggs for 24–36 h onto an agar plate topped with a small amount of heavy yeast paste. Adults were then discarded, and eggs were allowed to develop to adulthood at 25 °C in a vial capped at the open end by the plate. Heavy yeast paste was added to the plate throughout development as the sole food and nitrogen source. Virgin females were collected over CO_2_ within 8 h of eclosion and stored in a separate vial with ^15^N yeast paste on an agar plate. Shotgun MS analysis of proteins from whole, first-generation ^15^N flies was used to confirm isotopic labeling. In parallel to ^15^N labeling, males of the strain to be tested were grown in standard vials. Males were collected while young (0–3 d old) and aged in isolation in a standard vial.

### Mating experiments.

We performed 12 total mating experiments: three biological replicates each of Canton S and *tudor* males, and two biological replicates each of DTA-E, D. simulans, and D. yakuba males. For each experiment, males and females were aged to 2–5 d before mating. On the day before an experiment, approximately 40 labeled, virgin females were divided into three vials containing agar with a small amount of heavy yeast paste. Unlabeled males, in a ≥1.5-fold excess relative to females, were placed into three standard vials. The next day, males were transferred to the female vials without anesthesia. Mating was allowed to proceed for 2 h; vials were inspected several times during this period to confirm that copulations occurred. At the end of the mating period, flies were sexed over CO_2_: males were discarded, while females were kept on ice and immediately dissected in 50 mM ammonium bicarbonate. The lower female reproductive tracts were retained and stored in cold ammonium bicarbonate, while ovaries were excluded to prevent saturating the protein sample with the highly abundant egg yolk proteins. (If ovaries had been included, a greater fraction of peptides identified by MS would have arisen from these female proteins, making it more difficult to detect peptides from lower-abundance male Sfps.) It is unlikely that the removal of the ovaries diminished our ability to detect certain Sfps, as we identified all five Sfps (Acp26Aa, Acp36DE, Acp62F, msopa, and Spn2) that had been shown previously to localize to the ovaries [28].

### Protein preparation.

Because we sought to identify soluble, extracellular male Sfps, proteins were prepared in such a way so as to select specifically for soluble proteins. We also sought to reduce cell lysis and thus protein content from male sperm cells and female reproductive tract epithelial cells, since releasing intracellular proteins from these cells would dilute the concentration of transferred Sfps and render their identification more difficult. Female reproductive tracts were homogenized in the ammonium bicarbonate dissection buffer, which lacks any type of detergent and thus minimized cell lysis. The mixture was then centrifuged for 5 min at 18,000*g*. This process was repeated once, and the supernatant was retained. Protein concentration was estimated using a BCA assay (Pierce). Proteins were prepared for tandem mass spectrometry and digested with trypsin as previously described [57].

### Mass spectrometry.

Two samples each of Canton S and *tudor*, and one sample each of DTA-E, D. simulans, and D. yakuba, were analyzed by multi-dimensional protein identification technology (MudPIT) [58]. Protein digests (50 μg) were bomb-loaded overnight onto a tri-phasic 100-μm internal diameter capillary column packed with 15-cm reversed phase material (Jupiter C12, 4 μm, 90 Å; Phenomonex) at the tip of the column, then 4 cm of strong cation exchange material (Whatman), then 3 cm more of C12 material. The columns were then placed on-line with either an LTQ ion-trap mass spectrometer (ThermoElectron) or an LTQ-FT Ultra mass spectrometer (ThermoElectron) and eluted over a 12-step gradient with increasing salt concentration as described previously [59]. We also analyzed additional samples using a single reversed-phase HPLC method. One sample each of Canton S, *tudor*, DTA-E, D. simulans, and D. yakuba was analyzed with 75-μm internal diameter capillary columns packed with 40 cm of Jupiter C12 reversed phase material. For each sample analyzed by reversed phase, four or five technical replicates of ∼6 μg of protein were analyzed by injecting the sample directly into an on-line column and running four-hour gradients to acquire mass spectra using data-dependent acquisition.

### Protein identification.

Tandem mass spectra from each RAW mass spectrometry data file were extracted from the proprietary data format and stored in the MS2 file format [60] using in-house developed software. The charge-state of multiply charged MS/MS spectra were assigned a single +2 and +3 charge state using the *charge-czar* program [61] and searched against two databases using Sequest [62]. One database contained the annotated proteome of the appropriate species; the other database contained a set of “decoy” proteins, made by randomly shuffling the amino acids in each protein of the annotated database. Each database also included common contaminants (or their shuffled counterparts). For D. melanogaster samples, the proteome was taken from the version 4.3 release of the D. melanogaster genome (downloaded from NCBI; gene annotations and names were later updated to version 5.2). For D. simulans and D. yakuba, the GLEANR protein predictions from the 12-genome *Drosophila* sequencing project were used [35,36]. Because the GLEANR sets were likely imperfect, these species' databases were supplemented with the best hit (*e*-value cutoff = 0.01) obtained when the identified D. melanogaster proteins were searched using tblastn against the D. simulans or D. yakuba sequences in GenBank. After the database searches, the *percolator* program [32] was used to improve the discrimination between correct and incorrect peptide spectrum matches and to assign a *q*-value as a measure of the false discovery rate [63].

### Lists of transferred proteins (Tables S2 and S4).

To determine the list of high-confidence Sfps in D. melanogaster, we used the following criteria. Proteins identified in at least two independent experiments were automatically included. For proteins identified in only one of the six Canton S and *tudor* experiments, we required additional evidence that the protein could plausibly be involved in reproduction. This criterion could be satisfied if a protein was included in the most recent and comprehensive set of predicted Sfps [21] and/or if the protein showed strong evidence of being expressed exclusively or predominantly in the male reproductive tract (accessory glands or testes) in the FlyAtlas dataset [27]. Because we performed fewer mating experiments (two per species) and had no genome-wide catalog of Sfps or expression data, it was necessary to use different criteria in defining the sets of transferred proteins in D. simulans and D. yakuba. For each species, all proteins found in both experiments were automatically included. Furthermore, we included proteins found in only one experiment if they met any of the following criteria: (a) at least two peptides were used to identify the protein in the experiments; (b) if a single peptide was used for identification, it was detected at least twice during the MS run; or (c) the protein was identified as a transferred or predicted Sfp in D. melanogaster. After determining the list of transferred proteins shown in Table S2, functional information was acquired by examining FlyBase and the primary literature and was used to classify proteins listed in Table 1. We classified a protein as a “sperm protein” if it was found at least twice in Canton S experiments but not in *tudor* experiments, and/or if it was previously documented as such [25]. We used BLAST and BLAT searches to determine whether any transferred proteins of each species could be called as lineage-specific (Table S5).

### Genomic locations.

Genomic locations of Sfps were determined by downloading from FlyBase (release version 5.2) the chromosomal location of the first transcribed base of each gene, and recording the strand from which the gene was transcribed. Only euchromatic genes were considered and plotted, such that plots in Figure 2A do not indicate, for example, centromeric heterochromatin. Clusters were defined as genes that were within 10 kb of each other. For proteins encoded in a given cluster, we used pairwise BLASTP searches to determine whether the proteins showed evidence of paralogy. We used simulations to estimate a null distribution of the number of clusters that would be expected for a set of 138 genes distributed across the chromosomes in the same ratio as our Sfps. We extracted coding sequence annotations from http://www.flybase.org/ (version 5.2) and noted the location of the start codon for each gene (one isoform per locus). We then generated 1,000 sets of 138 genes by randomly selecting genes from each chromosome arm in the same ratio as the observed Sfps. The number of clusters in each set was counted; the median was 3, and the range was 0–9 clusters. Therefore, we judged our observed 19 clusters to be significantly more than would be expected by chance.

### RT-PCR.

Several GLEANR-predicted proteins identified in D. simulans and D. yakuba lacked annotated orthologs in the version 4.3 release of the D. melanogaster genome (one has since been annotated as CG12828, and another is reported [64] in GenBank under accession number BK003861, but is not yet recorded in FlyBase). We thus tested whether these genes (GLEANR numbers: dsim_2617, dsim_3447, dsim_15012, dsim_10234/dyak_792, dsim_9514/dyak_14199, dyak_12348, and dyak_10591) were expressed in D. melanogaster and showed sex-specific expression. PCR primers were designed to amplify transcripts in both the species of identification and the syntenic region of D. melanogaster. Although several of these proteins were encoded by short, single-exon genes, primers were designed to span putative introns when possible. Total RNA was prepared from whole male and whole female flies of both species using the TRIzol reagent (Invitrogen) and subjected to rigorous DNase treatment using the Turbo DNase kit (Ambion). First-strand cDNA from each sex was synthesized using the SuperScript III kit (Invitrogen) according to the manufacturer's instructions. This cDNA was then diluted and used in PCR reactions. As a positive control, we assayed for transcription of *ribosomal protein L32* (*RpL32*) using previously published primers [12], modified as needed for D. simulans and D. yakuba. Negative PCR controls were performed by using template from cDNA reactions that lacked reverse transcriptase.

### Evolutionary analyses.

For each D. melanogaster protein identified, we used coding-sequence alignments generated by the 12-species genome sequencing projects [35,36] to conduct molecular evolutionary analyses. We preferentially used the more recent Fuzzy Reciprocal BLAST-based alignments of D. melanogaster coding sequences with orthologs in any other species (ftp://ftp.flybase.net/genomes/12_species_analysis/clark_eisen/alignments); however, less than half of our Sfps were included in this set, so for the others we used the comparative assembly freeze 1 (CAF1) GeneMapper alignments produced by S. Chatterji and L. Pachter. From these sources, we were able to analyze 116 of the 138 annotated Sfps from D. melanogaster. We first made pairwise estimates of *d*_N_/*d*_S_ with model M0 of *codeml* in the PAML package [65]. When available, we used the D. simulans ortholog; otherwise, the D. sechellia ortholog was used. Alignments were obtained from one of the above sources and checked by eye using MEGA 4.0 [66]. For genes with pairwise *d*_N_/*d*_S_ ≥ 0.5 or *d*_N_ ≥ 0.05, we expanded our PAML analysis to up to five species (*melanogaster*, *simulans*, *sechellia*, *yakuba*, and *erecta*) in order to search for specific sites likely to have evolved under positive selection. For each gene, we used only those species for which alignments were reliable, and coding sequence alignments were checked by eye and edited in MEGA 4.0. We then tested for positive selection by comparing the likelihoods of *codeml* models M8 and M8a with a likelihood ratio test [67]. In model M8a, each codon is assigned to one of 11 classes, ten of which have an ω (*d*_N_/*d*_S_) value between 0 and 1 that is estimated from the data using maximum likelihood, and the 11th of which has ω = 1, representative of neutral evolution. Model M8 differs in that the 11th class of codons can take any ω value; this value is estimated from the data and can be greater than 1 (which indicates adaptive evolution). We corrected for multiple testing with a strict Bonferroni correction, though we note that among the 36 tests performed, only ∼2 would be expected to be false positives at a critical *p*-value of 0.05. As shown in Figure 2B and 2C, we re-analyzed *CG17472* and *CG31680* by including both paralogs from D. simulans and D. sechellia. The M8 versus M8a test of *CG17472* in the results section contains all orthologs (including both duplicate copies in D. simulans and D. sechellia), but not *CG31680* and its D. sechellia ortholog. For Figure 2C, we used the *dnaml* program in PHYLIP [68] to construct a phylogeny and to simulate 1,000 bootstrap replicates. We then used PAML to estimate ω for each branch of the phylogeny and performed a likelihood ratio test to compare the likelihoods of a model that allowed for ω to vary on each branch of the phylogeny versus a null model in which a uniform ω was estimated across all branches [69].

### Protein abundance.

Relative protein abundance was estimated from D. melanogaster MS data by counting the number of spectra that positively identified each protein in a given MS run. This spectral count was normalized for the length of each protein and divided by the sum of all normalized counts for the entire MS run to produce an NSAF for each protein, as previously described [38,39]. This value was then averaged across all experiments in which a protein was detected, and identified proteins were ranked by their mean NSAFs. This rank should be interpreted as how common it was to identify ionizable and detectable spectra for a given protein, relative to the other unlabeled proteins.

### Discovery of new, unannotated proteins.

To identify unannotated Sfps, we first used *nr6frame* (D. States, unpublished program) to make a six–reading-frame translation of the entire Berkeley *Drosophila* Genome Project D. melanogaster genome, version 5 (downloaded from ftp://hgdownload.cse.ucsc.edu/goldenPath/dm3/chromosomes). This program translates genomic DNA in all six reading frames; each reported ORF ends with a stop codon (but does not necessarily start with a methionine). Across the four *Drosophila* chromosomes, over 7.6 million ORFs were generated. We filtered these ORFs to exclude those that contained only one type of amino acid (mono-residue repeats), those that were too short to be confidently used in MS spectrum identification (<11 residues), or those that could not produce a tryptic peptide due to a lack of a K or R residue. This filtering reduced the data set to >5.8 million ORFs. For searching this large database, it was computationally advantageous to filter the MS2 files in order to reduce the search time. We used data from three technical replicates of a *tudor* sample, collected with a 40-cm reversed phase column on an LTQ-FT Ultra instrument. We used Hardklör [40] to predict the isotope distributions that resulted from ^15^N-enriched peptides and removed their corresponding MS/MS spectra from the analysis. Because of the excess of labeled peptides within the sample, this filtering reduced the number of spectra that needed to be searched by ∼86%. The remaining spectra were then searched against the six-frame translation database using Sequest [62], and identifications were filtered by DTASelect. Identified peptides matching annotated protein coding genes were discarded, leaving 23 ORFs that did not match a genome annotation. We designed primers in the genomic regions matching the identified peptides and performed 5′ and 3′ RACE to amplify transcripts from these regions (SMART RACE Kit, Clontech-Takara). This method identified 19 unannotated genes, which were then confirmed with RT-PCR and sequencing of cDNA from whole males. SignalP was used to predict whether each novel protein is secreted [70], and we used BLAST and PHYRE [71] searches to determine whether any protein had sequence or structural homology to other proteins.

## Supporting Information

Figure S1 Comparison of Sfps Detected in D. melanogaster, D. simulans, and D. yakuba
(164 KB PDF)

Table S1 Proteins Identified in Two DTA-E Mating Experiments (66 KB DOC)

Table S2 High-Confidence Transferred Sfps Detected in Canton S and *tudor* Experiments (58 KB XLS)

Table S3 Multiple-Species PAML Analyses of Sfps with Elevated *d*_N_/*d*_S_ or *d*_N_ in Pairwise Comparisons (41 KB XLS)

Table S4 High-Confidence Transferred Sfps Detected in D. simulans and D. yakuba
(26 KB XLS)

Table S5 Lineage-Specific Gene Gain and Loss Events for Proteins Detected in Only One or Two Species Analyzed (43 KB DOC)

Table S6 Novel D. melanogaster Genes Identified by a Six-Frame Translation Database Search and Confirmed by RACE and RT-PCR (161 KB DOC)

### Accession Numbers

Sequence data has been deposited in GenBank under accession numbers EU755332–EU755350.

## Abbreviations

Acp: accessory gland protein
EST: expressed sequence tag
MS: mass spectrometry
NSAF: normalized spectral abundance factor
Obp: odorant binding protein
ORF: open reading frame
RACE: rapid amplification of cDNA ends
RT-PCR: reverse transcriptase polymerase chain reaction
Sfp: seminal fluid protein
