# Supplementary figures and images for: Proteomics Reveals Novel Drosophila Seminal Fluid Proteins Transferred at Mating
Source: PLoS Biol. 2008 Jul 29;6(7):e178. doi: 10.1371/journal.pbio.0060178 (PMC2486302; doi:10.1371/journal.pbio.0060178)

*D. melanogaster* (138)

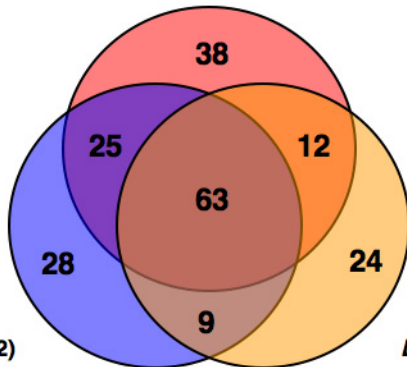

*D. simulans* (122)

*D. yakuba* (107)

Supplement: Figure S1 — (164 KB PDF) [file pbio.0060178.sg001.pdf]
