# Supplementary material for: Proteomics Reveals Novel Drosophila Seminal Fluid Proteins Transferred at Mating
Source: PLoS Biol. 2008 Jul 29;6(7):e178. doi: 10.1371/journal.pbio.0060178 (PMC2486302; doi:10.1371/journal.pbio.0060178)
Supplement: Table S1 — (66 KB DOC) [file pbio.0060178.st001.doc]

**Table S1. Proteins detected when mating DTA-E males to ^15^N-labeled females.**

| **Protein Name** | **CG Number** | **FBgn Number** | **# Exp** | **Other Information** |
| --- | --- | --- | --- | --- |
| CG11041 | CG11041 | FBgn0034481 | 1 | FlyAtlas shows little or no expression in testes and accessory glands [1] |
| CG17242 | CG17242 | FBgn0042188 | 1 | FlyAtlas suggests accessory gland expression [1] |
| CG31704 | CG31704 | FBgn0051704 | 1 | FlyAtlas suggests accessory gland expression [1] |
| CG6289 | CG6289 | FBgn0036970 | 2 | FlyAtlas suggests accessory gland expression [1] |
| Cpr67Fb | CG18348 | FBgn0036110 | 2 | FlyAtlas shows little or no expression in testes and accessory glands [1] |
| Est-6 | CG6917 | FBgn0000592 | 1 | Known ejaculatory duct protein [2] |
| lectin-46Ca | CG1656 | FBgn0040093 | 1 | FlyAtlas suggests accessory gland expression [1] |
| Met75-Ca/  Met75-Cb | CG32197/  CG18064 | FBgn0028416/  FBgn0028415 | 2 | Met75-Ca and -Cb encode identical proteins; reported in GenBank to encode ejaculatory duct proteins (accession AJ249253) |
| Obp56g | CG13873 | FBgn0034474 | 2 | FlyAtlas suggests accessory gland expression [1] |
| Peb | CG2668 | FBgn0004181 | 1 | Known ejaculatory bulb protein [3] |
| PebII | CG2665 | FBgn0011694 | 2 | Known ejaculatory bulb protein [4] |

Notes: # Exp = number of experiments (out of 2) in which the protein was detected. Proteins showing accessory gland expression in FlyAtlas may be produced in the secondary cells of the accessory glands, which are not affected by the DTA-E background [5].

References

1. Chintapalli VR, Wang J, Dow JAT (2007) Using FlyAtlas to identify better *Drosophila melanogaster* models of human disease. Nat Genet 39: 715-720.

2. Mane SD, Tompkins L, Richmond RC (1983) Male esterase 6 catalyzes the synthesis of a sex pheromone in Drosophila melanogaster females. Science 222: 419-421.

3. Lung O, Wolfner MF (2001) Identification and characterization of the major *Drosophila melanogaster* mating plug protein. Insect Biochem Mol Biol 31: 543-551.

4. Dyanov HM, Dzitoeva SG (1995) Method for attachment of microscopic preparations on glass for in situ hybridization, PRINS and in situ PCR studies. Biotechniques 18: 822-826.

5. Kalb JM, DiBenedetto AJ, Wolfner MF (1993) Probing the function of *Drosophila melanogaster* accessory glands by directed cell ablation. Proc Natl Acad Sci U S A 90: 8093-8097.
