# Supplementary material for: Proteomics Reveals Novel Drosophila Seminal Fluid Proteins Transferred at Mating
Source: PLoS Biol. 2008 Jul 29;6(7):e178. doi: 10.1371/journal.pbio.0060178 (PMC2486302; doi:10.1371/journal.pbio.0060178)
Supplement: Table S5 — (43 KB DOC) [file pbio.0060178.st005.doc]

**Table S5. Seminal fluid proteins found with MS in only one or two species for which an ortholog was not easily identifiable in other species.**

***Detected only in* D. melanogaster**

Gene Comments

CG31680* Appears to be unique to *Dmel* and *D. sechellia*, but in a repetitive region of the genome

CG6663* Lineage-specific duplication of CG6289

msopa Found in *Dsim* ESTs (Swanson et al. 2001), but gap in the *Dsim* genome sequence prevented gene prediction; absent in *Dyak*

***Detected only in* D. simulans**

Gene Comments

dsim_GLEANR_6950* Appears to be lineage specific; no strong BLAST or BLAT hit in other species; shows homology with *Dmel* lectins

dsim_GLEANR_15012 Mispredicted gene structure in *Dsim*; actually a short, 105-nt gene that was found in our **sixframe** search for *Dmel*

dsim_GLEANR_1947* Unannotated in *Dmel* and not predicted in *Dyak*, but the *Dmel* region appears to encode a full-length polypeptide

dsim_GLEANR_2617 Corresponding *Dmel* protein found with **sixframe** search; *Dyak* homolog predicted by Begun et al. (2006) EST sequencing

dsim_GLEANR_3447 Not annotated in *Dmel*, but full-length ORF appears in tact and was detected with RT-PCR

***Detected only in* D. yakuba**

Gene Comments

dyak_GLEANR_2330* Syntenic region of DNA can be found in *Dmel* and *Dsim*, but no annotated genes present

dyak_GLEANR_2331* Lineage-specific duplication of dyak_GLEANR_2330; no syntenic region identifiable in *Dmel* and *Dsim*

dyak_GLEANR_6542* Lineage-specific duplication of Acp76A

dyak_GLEANR_19266* Lineage-specific duplication of CG16713

dyak_GLEANR_10591* Psuedogenized in *Dsim*, not present in *Dmel*

dyak_GLEANR_12348 Unannotated in *Dmel*; detected with RT-PCR but not in sixframe search

***Detected in* D. melanogaster *and* D. simulans**

Gene Comments

Acp24A4* Lineage-specific duplication in *Dmel/Dsim* or loss in *Dyak*

Acp29AB* Lineage-specific duplication of lectin-29Ab in *Dmel*/*Dsim*

Acp63F* Short protein, probably lineage-specific to *Dmel*/*Dsim*

Acp95EF Transcripts reported in *Dyak* (Begun et al.), so may be incomplete sequence or incorrect prediction

CG15635 Highly repetitive sequence, rendering alignments and conclusions difficult

Mst57Dc* Syntenic region present in *Dyak*; probably lineage-specific to *Dmel*/*Dsim*

***Detected in* D. melanogaster *and* D. yakuba**

Gene Comments

Anp *Dsim* aligns well but is missing the stop codon

CG32883 Incorrect assembly in *Dsim* (half of the gene is on chr 2R_random)

CG5267 Start codon mutated to ATA in *Dsim* (or sequence error)

Obp22a Gap in the *Dsim* assembly

Spn3* Not predicted in *Dsim* GLEANR set, though syntenic region present

***Detected in* D. simulans *and* D. yakuba**

Gene Comments

dyak_GLEANR_792/ Unannotated in *Dmel* v. 4.3; now called CG12828; found by

dsim_GLEANR_10234 RT-PCR

dyak_GLEANR_14199/ Unannotated in *Dmel*, but predicted by Hild et al. (2003); found

dsim_GLEANR_9514 in **sixframe** search

*Indicates genes that are likely to be restricted to one or two lineages.
