# Supplementary material for: Proteomics Reveals Novel Drosophila Seminal Fluid Proteins Transferred at Mating
Source: PLoS Biol. 2008 Jul 29;6(7):e178. doi: 10.1371/journal.pbio.0060178 (PMC2486302; doi:10.1371/journal.pbio.0060178)
Supplement: Table S6 — (161 KB DOC) [file pbio.0060178.st006.doc]

**Table S6. Previously unannotated, experimentally verified seminal fluid proteins in *D. melanogaster*.**

| **Name** | **Location*** | **Length (a.a.)** | **Annotated Sfps Nearby** | **BLASTP Similiarity**  **(*e* < 0.01)‡** | **PHYRE Similarity**  **(*e* < 0.01)** |
| --- | --- | --- | --- | --- | --- |
| Sfp23F | Chr2L: 3387708-3387642, 3387573-3387404 | 78 | none | CG16704 (**serine protease inhibitor**) | n.s. |
| Sfp24Ba | Chr2L: 3668531-3668471, 3668417-3668113 | 121 | Acp24A4 | beta-bungarotoxin | bikunin (**Kunitz-type protease inhibitor**) |
| Sfp24Bb | Chr2L: 3669709-3669681, 3669585-3669318 | 100 | Acp24A4 | n.s. | n.s. |
| Sfp24C1 | Chr2L: 3700700-3700763, 3700820-3701045 | 96 | Acp24A4 | n.s. | n.s. |
| Sfp24F | Chr2L: 4442051-4441976, 4441921-4441470 | 175 | none | CG9978 (**galactose-specific C-type lectin**) | **C-type lectin** |
| Sfp26Ac | Chr2L: 5884751-5884730, 5884676-5884507, 5884441-5884424 | 84 | CG9029, Acp26Aa, Acp26Ab | n.s. | n.s. |
| Sfp33A1 | Chr2L: 11754479-11754404, 11754344-11754139 | 93 | none | n.s. | n.s. |
| Sfp33A2 | Chr2L: 11837729-11837881 | 50 | CG31704, CG31758 | n.s. | n.s. |
| Sfp33A3 | Chr2L: 11839935-11840234 | 99 | CG31704, CG31758 | CG31704 | **hydrolase inhibitor** |
| Sfp35C | Chr2L: 15202022-15202038, 15201984-15201822, 15201762-15201707 | 81 | none | *Dpse*_GA21004 | n.s. |
| Sfp51E | Chr2R: 11080726-11080573, 11080509-11080244 | 139 | none | n.s. | n.s. |
| Sfp53D | Chr2R: 12632590-12632111 | 159 | Acp53C14a,b,c, Acp53Ea, CG5267 | TPA_inf: HDC06796, *Dsim*_9514, *Dyak*_14199 | n.s. |
| Sfp60F | Chr2R: 21060161-21060147, 21060094-21059918 | 63 | PebII, Peb | n.s. | n.s. |
| Sfp65A | Chr3L: 6343410-6343835 | 141 | none | n.s. | n.s. |
| Sfp70A4 | Chr3L: 13289956-13289789 | 55 | Acp70A | n.s. | n.s. |
| Sfp77F | Chr3L: 20890498-20890420, 20890362-20890193 | 82 | none | n.s. | n.s. |
| Sfp79B | Chr3L: 22073439-22073546 | 35 | msopa | *Dsim*_15012 | n.s. |
| Sfp84E | Chr3R: 3806011-3806013, 3805962-3805483 | 160 | none | n.s. | n.s. |
| Sfp87B | Chr3R: 8098902-8099165 | 87 | none | GH21617p, *Dsim*_2617 | n.s. |

*Coding DNA sequences have been deposited in GenBank under accession numbers EU755332–EU755350 and submitted to FlyBase. In cases where the first exon appears short, additional 5’ UTR sequence was obtained by RACE, lending high confidence to the transcript.

**‡** *Dsim* and *Dyak* numbers refer to GLEANR predicted protein numbers; *Dpse* refers to annotated *D. pseudoobscura* proteins. Other numbers refer to GenBank entries that are not annotated in FlyBase.
